# A markov classification model for metabolic pathways

**DOI:** 10.1186/1748-7188-5-10

**Published:** 2010-01-04

**Authors:** Timothy Hancock, Hiroshi Mamitsuka

**Affiliations:** 1 Bioinformatics Center, Institute for Chemical Research, Kyoto University, Japan

## Abstract

**Background:**

This paper considers the problem of identifying pathways through metabolic networks that relate to a specific biological response. Our proposed model, HME3M, first identifies frequently traversed network paths using a Markov mixture model. Then by employing a hierarchical mixture of experts, separate classifiers are built using information specific to each path and combined into an ensemble prediction for the response.

**Results:**

We compared the performance of HME3M with logistic regression and support vector machines (SVM) for both simulated pathways and on two metabolic networks, glycolysis and the pentose phosphate pathway for *Arabidopsis thaliana*. We use AltGenExpress microarray data and focus on the pathway differences in the developmental stages and stress responses of *Arabidopsis*. The results clearly show that HME3M outperformed the comparison methods in the presence of increasing network complexity and pathway noise. Furthermore an analysis of the paths identified by HME3M for each metabolic network confirmed known biological responses of *Arabidopsis*.

**Conclusions:**

This paper clearly shows HME3M to be an accurate and robust method for classifying metabolic pathways. HME3M is shown to outperform all comparison methods and further is capable of identifying known biologically active pathways within microarray data.

## Background

Networks are a natural way of understanding complex processes involving interactions between many variables. Visualizing a process as a network allows the researcher to form an intuitive understanding of complex phenomena. A clear example of the effective use of networks is the visualization of metabolic networks to provide a detailed map of key chemical reactions and their genetic dependencies that occur within a cell. However the size and complexity of metabolic networks has increased to the point where the ability to understand the entire network is lost. Researchers must now rely on models of the network structure to capture the key functional components that relate to an observed response. In this paper we propose a model capable of identifying the key pathways through metabolic networks that are related to a specific biological response.

Metabolic networks, as described in databases such as KEGG [1], can be represented as directed graphs, with the vertices denoting the compounds and the edges labeled by the reactions. The reactions within metabolic networks are catalyzed by specific genes. If a gene is active, then it is possible for the corresponding reaction to occur. If a reaction is active then a pathway is created between two metabolic compounds that is labeled by the gene that catalyzed the reaction. Information about the activity of genes within metabolic networks can be readily obtained from microarray experiments. Microarray experiments are then used to view differences in gene activity under varying experimental conditions such as (**y **= **1**) patients treated with drug A and (**y **= **2**) patients treated with drug B. The question asked by such experiments is: are there any gene pathways that are differentially expressed when patients are given drug A or B? The abundance of publicly available microarray expression observations found in databases such as ArrayExpress [2] along with the detailed biological knowledge contained within pathway databases like KEGG, has spurred biologists to want to combine these two sources of information and model the metabolic network dynamics under different experimental conditions.

This paper proposes a novel classification model for identifying frequently observed paths within a specified network structure that can be used to classify known response classes. Our proposed model is a probabilistic combination of a Markov mixture model which identifies frequently observed pathway clusters and an ensemble of supervised techniques each trained locally within each pathway cluster to classify the response. We require the prior specification of the metabolic network, gene expression data and response variable that labels the experimental conditions of interest.

**Figure 1:**
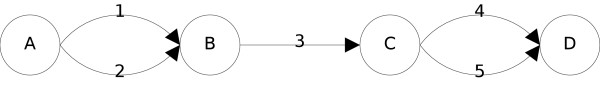
**Example network**.

To construct our model we consider the network to be a directed graph and pathways through the network to be binary strings. For example there are 4 possible paths between nodes A and D in the network described in Figure 1. In Figure 1 the binary representation of the path between A and D that traverses edges [1,3,4] is [1, 0,1, 1, 0]. If we interpret Figure 1 to be a metabolic network where the edges are the genes and the nodes are the compounds, then which paths are taken at any given time can be seen to be dependent on the activity of specific genes. If a gene is active, then it is possible to proceed along that edge within the network. In our experiments we extract all valid pathways from each microarray experiment that are observed between prespecified start and end compounds. To do this we treat each microarray experiment, *x*_*i *_as a single observation of the activity of all genes within a network. For each *x*_*i *_we also have a response label *y*_*i *_denoting the experimental conditions. Then defining an active edge to be an over-expressed gene observation within *x*_*i *_we extract all possible paths from the start node to the end node and label each path with *y*_*i*_. The resulting pathway dataset then consists of *N *observed paths from each microarray experiment each with a response label indicating the observed experimental group. Common bioinformatics solutions to this problem include using data mining techniques to classify the response based on the gene expression information and then overlay the finding on the metabolic pathway [3]. Although this approach can classify the response accurately, they use no knowledge of the network structure. Network structures can be incorporated into standard methods by defining an appropriate similarity measure between sequences and then employ a kernel technique, such as Support Vector Machines (SVM) [4] to classify the response. However, the specification of a similarity measure or kernel removes any ability to observe individual pathways and determine if the model identifies a meaningful biological result. An accurate classifier with the capability to extract the dominant pathways is required for a complete solution.

Graphical methods such as Bayesian networks present a framework capable of modeling a network structure imposed upon a dataset [5]. Bayesian networks search for the most likely network configuration by drawing edges connecting dependent variables. However, when considering mining the dominant paths within a known network such an approach may not be the most direct solution. For example constructing a Bayesian network of a metabolic pathway will join related genes by assuming a conditional dependence between each gene and its parent genes within the network. This dependency is valid when considering problems concerning the prediction of unknown structure [6,7] though may be inappropriate for the prediction of frequently observed paths through a known network structure. To predict frequently observed paths, a more natural assumption is accommodated by Markov methods which assume that the decision on the next step taken along a path only requires information on the current and next set of genes within the network.

Hidden Markov Models (HMM) are commonly used for identifying structure within sequence information [8]. HMMs assume that the nodes of the network are unknown and the observed sequences are a direct result of transition between these hidden states. However, if the network structure is known, a more direct approach is available through a mixture of Markov chains. Markov mixture models such as 3M [9] directly search for dominant pathways within sequence data by assuming each mixture component is a Markov chain through a known network structure. For metabolic networks, Markov mixture models, such as 3M, have been shown to provide an accurate and highly interpretable model of dominant pathways throughout a known network structure. However, both HMM and 3M are unsupervised models and therefore are not able to direct their search to explicitly uncover pathways that relate to specific experimental conditions.

The creation of a supervised classification technique that exploits the intuitive nature of Markov mixture models would be a powerful interpretable tool for biologists to analyze network pathways. In this paper we propose a supervised version of the 3M model using the Hierarchical Mixture of Experts (HME) framework [10]. We choose the mixture of experts framework as our supervised model because it provides a complete probabilistic framework for localizing a classification model to specific clusters within a dataset. Our proposed model, called HME3M employs a **HME **to combine the **3M **with penalized logistic regressions classifiers as the experts within each cluster to classify the response.

## Experiments

Our problem has the following inputs: the network structure, microarray observations and a response variable. A pathway through the network, *x*_*i*_, is assumed to be a binary vector, where a 1 indicates a traversed edge and 0 represents a non-traversed edge. The decision on which edges can be traversed is made for each microarray observation based on the expression of each gene. Once the set of valid edges have been defined, for each microarray observation all valid pathways are extracted. After extracting all observed pathways we label each path with the response label of the original microarray experiment. Once this is completed for all observations it is possible to set up a supervised classification problem where the response vector *y *denotes the response label of each pathway, and the predictor matrix *X *is an *N *× *P *binary matrix of pathways, where *N *is the number of pathways and *P *is the number of edges within the network. The binary predictor matrix, *X *and its response *y *can now be directly analyzed by our proposed pathway classifier, HME3M, and also with standard supervised techniques. We assess the performance of HME3M in both simulated and real data environments and compare it to PLR and Support Vector Machines (SVM) with three types of kernels, linear, polynomial (degree = 3) and radial basis. The implementation of SVM used for these experiments is sourced from the R package *e1071 *[11].

We point out here that the predictor matrix *X *is a list of all pathways through the network observed within the original dataset. Therefore *X *contains all available information on the given network structure contained within the original dataset. Using this information as input into the PLR and SVM models is supplying these methods with the same network information that is provided to the HME3M model. As the supplied information is the same for all models the comparison is fair. The performance of the models are expected to differ because SVM and PLR do not consider the Markov nature of the input pathways whereas HME3M explicitly models this property with a first order Markov mixture model.

Experiments comparing HME3M to standard classification techniques are performed first on simulated network pathways and then on real metabolic pathways and microarray expression data. We now describe the details of each experiment.

### Synthetic Data

To construct the simulation experiments we assume that the dataset is comprised of dominant pathways that define the groups and random noise pathways. To ensure that the pathway structure is the major information within the dataset, we specify the network structure and simulate only the binary pathway information. A dominant pathway is defined as a frequently observed path within a response class. The level of expression of a dominant pathway is defined to be the number of times it is observed within a group. A noise pathway is defined to be a valid pathway within the network that leads from the start to the end compounds but is not any of the specified dominant pathways. As the percent of noise increases, the relative expression of the dominant paths decreases, making correct classification harder.

**Figure 2:**
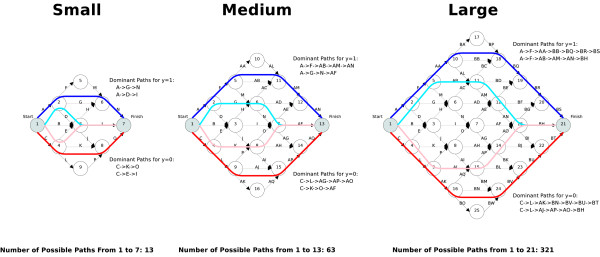
**Simulated network diagrams**.

We run the simulation experiments on three graphs with the same structure but with increasing complexities as shown in Figure 2. For each network we define two dominant pathways for each response label, *y *= 0 and *y *= 1 and give each dominant pathway equal pathway expression levels. We simulate a total of 200 pathways per response label which includes observations from the two dominant pathways and noise pathways. Separate simulations are then performed for the specified noise pathway percentages [10, 20, 30, 40, 50]. The performance of each method is evaluated with 10 runs of 10-fold cross-validation. The performance differences between HME3M compared to SVM and PLR are then tested with paired sample *t*-tests using the test set performances from the cross-validation. We set the HME3M parameters to be *M *= [2,3], *λ *= 1, *α *= 0.5.

### KEGG Networks

**Figure 3:**
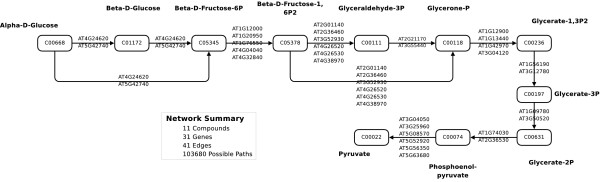
**Arabidopsis thaliana glycolysis pathway from Alpha-D-Glucose to Pyruvate**. For visual simplicity, we show only a single edge connecting each compound; however in the actual network there is a separate edge for each gene label displayed.

**Figure 4:**
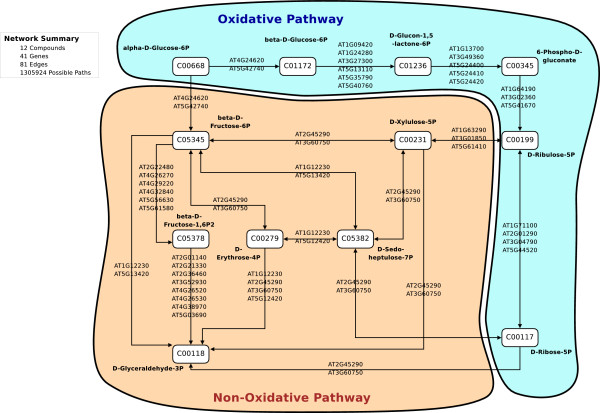
**Arabidopsis thaliana Oxidative Pentose Phosphate Cycle**. For visual simplicity, we show only a single edge connecting each compound; however in the actual network there is a separate edge for each gene label displayed.

To assess the performance of HME3M in a realistic we use two different metabolic networks both extracted from KEGG [1] for the *Arabidopsis thaliana *plant. The networks are selected for their differing structure and complexity. We deliberately use *Arabidopsis *as it has become a benchmark organism and it is well known that during the developmental stages and under stress conditions, different components of core metabolic pathways are activated. The first is glycoloysis (Figure 3) which is a simple left to right style network and the second is the pentose phosphate pathway (Figure 4) which is a simple directed cycle. Due to the large number of paths extracted for the KEGG networks to assess the performance of HME3M we conduct 20-fold inverse cross-validation for model sizes *M *= 2 to *M *= 10. Inverse 20-fold cross-validation firstly divides the observations randomly into 20 groups and then for each group trains using only observations from one group and tests the performance on the observations from the other 19. The performance of HME3M for 20-fold inverse cross-validation is compared to PLR and the SVM models.

#### KEGG Arabidopsis Glycolysis Pathway

In Figure 3 we extract from KEGG the core component of the glycolysis network for *Arabidopsis *between C00668 (*Alpha-D-Glucose*) and C00022 (*Pyruvate*). The extracted network in Figure 3 is a significantly more complex graph than our simulated designs and has 103680 possible pathways between C00668 and C00022. We extract the gene expression observations for all genes on this pathway from the AltGenExpress development series microarray expression data [12] downloaded from the ArrayExpress database [2]. The AltGenExpress development database [12] is a microarray expression record of each stage within the growth cycle of *Arabidopsis *and contains expression observations of 22814 genes over 79 replicated conditions. For our purposes we extract observations for *"rosette leaf" *(*n *= 21) and *"flower" *(*n *= 15) and specify "flower" to be target class (*y *= 1) and "rosette leaf" to be the comparison class (*y *= 0). For the glycolysis experiment we set the HME3M parameters to be: *λ *= 1 and *α *= 0.7.

To extract binary instances of the glycolysis pathway within our extracted data we scale the observations to have a mean of zero and standard deviation of 1. After scaling the expression denote active genes within the network using three tolerances [-0.1, 0, 0.1] and construct three separate datasets. Within each dataset we set any gene expression observation that is above the specified tolerance to be "1" or overexpressed, otherwise we set its value to "0" or underexpressed. The structure of each pathway dataset is presented in Table 1. This is a simple discretization as it requires no additional information from the response or external conditions that might limit the number of paths selected. We deliberately choose this simple discretization of the gene expressions as it provides a highly noisy scenario to test the performance of HME3M.

**Table 1:** Number of pathways extracted for the Arabidopsis glycolysis network for each gene activity tolerance.

Expression Tolerance	Flower Pathways	Rosette Leaf Pathways	Total
**-0.1**	12720	32664	**45384**
**0**	4288	20608	**24896**
**0.1**	3024	14496	**17520**

#### KEGG Arabidopsis Pentose Phosphate Pathway

In Figure 4 we extract from KEGG the core component of the pentose phosphate network for *Arabidopsis *between C00668 (*Alpha-D-Glucose*) and C00118 (*D-Glyceraldehyde 3-Phosphate*). The extracted network is more complex again than the glycolysis network and has 1305924 possible pathways between C00668 and C00118. We extract the gene expression observations for all genes on this pathway from the AltGenExpress abiotic stress microarray expression data [13].

The AltGenExpress abiotic stress database [12] contains gene expression measurements on the responses of the "Shoots" or "Roots" of *Arabidopsis *to various stress stimuli. For our purposes we extract observations for *Arabidopsis *"Shoots" in both the oxidative stress and control groups for all observed times from 0.25 to 3 hours. This results in six experiments from the *"Oxidative" *(*n *= 6) and 10 experiments from the *"Control" *(*n *= 10) and we specify "Oxidative" to be target class (*y *= 1) and "Control" to be the comparison class (*y *= 0).

We select this particular subset of the AltGenExpress abiotic stress as observations on the metabolite abundance for the pentose phosphate pathway [14] clearly show that within the first 3 hours of exposure to oxidative stress a significant increase in the abundance of C00117 (D-Ribose 5-phosphate) is observed. In [14] it was suggested that this increase was a result of an increase in the flux through the oxidative branch of the pentose phosphate pathway (Figure 4). In this paper we try to confirm this observation within the AltGenExpress abiotic stress with HME3M.

To extract binary instances of the pentose phosphate network within our extracted data we scale the observations to have a mean of zero and standard deviation of 1. After scaling the expression denote active genes within the network using three tolerances [0, 0.05, 0.1] and construct three separate datasets. The structure of each pathway dataset is presented in Table 2. We use different tolerances to the glycolysis pathway experiments due to the excessively large number of pathways extracted for negative tolerance values Table 2. For the pentose phosphate experiment we set the HME3M parameters to be: *λ *= 2 and *α *= 1.

**Table 2:** Number of pathways extracted for the Arabidopsis pentose phosphate network for each gene activity tolerance.

Expression Tolerance	Oxidative Pathways	Control Pathways	Total
**-0.1**	157468	48557	**206025**
**-0.05**	84503	44320	**128823**
**0**	19225	43777	**63002**
**0.05**	42846	14422	**57268**
**0.1**	10086	8935	**19021**

## Results and Discussion

### Synthetic Data

For the synthetic data the correct classification rate (CCR) percentages, ranges and paired sample *t*-test results for simulated graphs are shown in Table 3. All experiments show HME3M outperforming the trialled SVM kernels and a single PLR model. In fact, the only times when the performances of SVM and HME3M are equivalent (*P*-value < 0.05) is with the small or medium graph with high levels of within group noise. Of particular note is the observation that for the medium and large graphs the median performance for HME3M is always superior to SVM. Furthermore, as the graph complexity increases it is clearly seen that HME3M consistently outperforms SVM and this performance is maintained despite the increases in the percent of noise pathways.

**Table 3:** The median and range of the 10 × 10-fold correct classification rates (CCR) for all simulation experiments.

		Percent Within Group Noise									
Graph	Model	*M *= 2					*M *= 3				
		0.1	0.2	0.3	0.4	0.5	0.1	0.2	0.3	0.4	0.5
**Small**	HME3M	**96.79**	**90.94**	**85.79**	**79.80**	74.97	**96.14**	**92.64**	**86.46**	**79.86**	**76.13**
	**CCR Range**	4.15	6.99	4.49	7.42	6.73	3.36	7.69	3.98	16.83	7.36
	PLR	50.67*	50.98*	50.68*	50.35*	50.96*	50.83*	50.70*	50.58*	50.62*	50.70*
	**CCR Range**	1.53	1.54	2.13	1.66	1.55	1.11	1.71	1.35	1.42	0.68
	SVM (linear)	95.14*	90.48*	85.28	78.94	72.53*	94.77*	89.01*	85.16*	78.66	75.21
	**CCR Range**	0.72	2.31	3.16	2.61	7.02	1.61	4.39	2.72	11.55	14.54
	SVM (polynomial)	95.25*	90.05*	84.19*	79.42	75.03	94.91*	89.71*	84.82*	78.66	75.73
	**CCR Range**	1.52	1.18	3.74	3.15	6.92	2.33	3.67	4.38	3.09	8.41
	SVM (radial)	95.28*	89.73*	84.19*	79.39	**75.07**	94.37	89.48*	84.82*	78.94	75.35
	**CCR Range**	1.53	2.81	4.20	2.61	8.02	2.32	4.53	3.28	4.14	7.29

**Medium**	HME3M	**98.94**	**94.29**	**88.68**	**80.78**	**77.05**	**98.80**	**96.22**	**90.11**	**84.02**	**77.89**
	**CCR Range**	2.92	6.18	5.73	12.51	5.56	2.40	5.59	10.57	8.98	8.76
	PLR	50.48*	50.62*	50.68*	50.48*	50.55*	50.55*	50.35*	50.62*	50.55*	50.48*
	**CCR Range**	2.04	0.99	1.30	2.13	1.23	0.97	0.69	1.63	1.31	1.13
	SVM (linear)	94.68*	89.80*	83.91	79.64*	76.02	94.28*	89.52*	84.80*	80.19*	73.41*
	**CCR Range**	2.74	22.91	3.56	4.17	4.12	1.72	2.60	5.17	2.89	5.40
	SVM (polynomial)	94.92*	90.30*	84.53*	79.32*	75.07*	94.22*	90.35*	84.53*	79.72*	75.21*
	**CCR Range**	2.73	21.22	4.00	5.80	2.83	1.65	3.69	6.85	2.85	4.91
	SVM (radial)	94.60*	90.30*	84.52*	79.28*	74.70*	94.19*	89.68*	84.31*	80.72*	74.80*
	**CCR Range**	2.45	4.35	2.82	2.99	4.56	1.90	3.49	4.67	2.93	3.59

**Large**	HME3M	**99.54**	**97.36**	**93.29**	**83.73**	**77.88**	**99.39**	**97.52**	**94.08**	**84.78**	**83.11**
	**CCR Range**	2.96	5.00	9.44	10.17	9.97	1.77	6.12	10.23	10.05	11.56
	PLR	50.69*	50.27*	50.49*	50.56*	50.62*	50.41*	50.70*	50.34*	50.83*	50.82*
	**CCR Range**	1.40	1.27	1.69	1.69	0.82	1.11	1.39	1.51	0.98	1.09
	SVM (linear)	94.91*	89.31*	85.58*	79.55*	73.79*	94.23*	89.34*	85.30*	79.67*	74.34*
	**CCR Range**	0.76	3.29	4.66	3.72	4.51	2.24	2.18	2.68	2.77	7.30
	SVM (polynomial)	94.78*	89.45*	85.80*	78.67*	74.05*	94.28*	89.86*	84.69*	79.44*	74.33*
	**CCR Range**	2.22	2.87	4.94	3.46	4.60	2.24	15.39	3.24	4.66	4.86
	SVM (radial)	94.98*	89.74*	85.09*	79.36*	74.21*	94.62*	89.83*	84.47*	79.56*	74.62*
	**CCR Range**	1.43	4.00	5.29	2.62	2.66	1.69	2.39	2.28	4.99	6.10

The best model performances are emboldened and a * flags models performances that are significantly different from HME3M (*α *= 0.05).

The performance of PLR for the simulated pathways is particularly poor because the dataset is noisy and binary. PLR can only optimize on these noisy binary variables and is supplied with no additional information such as the kernels of the SVM models and the pathway information of HME3M. Additionally, the L2 ridge penalty is not a severe regularization and will estimate coefficients for pure noise pathway edges. Combining the lack of information within the raw binary variables with the nature of L2 regularization, it is clear in this case that PLR will overfit and lead to poor performance.

Table 3 also demonstrates that as you increase the number of mixture components in the HME3M model, *M*, the model's resistance to noise increases. The increased robustness of HME3M is observed in the increase in median performance from *M *= 2 to *M *= 3 when the noise levels are 30% or more (≥ 0.3). A supporting observation of particular note is that when the performances of HME3M with *M *= 2 is compared with the linear kernel SVM on the medium graph and 50% noise there is no significant difference between the model's performances. However, by increasing *M *to 3, HME3M is observed to significantly outperform linear kernel SVM. Further, in a similar but less significant case, for the small graph with 50% added noise, by increasing *M *from 2 to 3 the median performance of HME3M becomes greater than that of linear kernel SVM. Although this increase did not prove to be significant the observed increasing trend within the median performance is clearly driving the results of the *t*-test.

It is noticeable in Table 3 that the HME3M performance can be less precise than SVM or PLR models. However the larger range of CCR performances is not large enough to affect the significance of the performance gains made by HME3M. The imprecision of HME3M in this case is most likely due to the constant specification of *λ*, *α *and *M *over the course of the simulations. In the microarray data experiments we show that careful choice of *M *produces stable model performances with a comparable CCR range than the nearest SVM competitor.

### KEGG Arabidopsis Glycolysis Pathway

**Figure 5:**
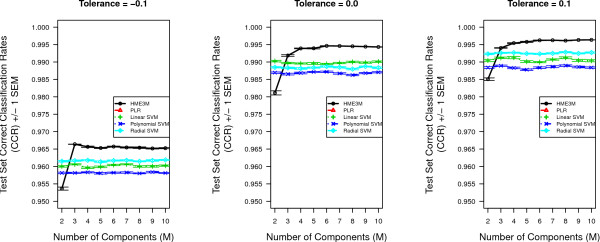
**Performance results for the Glycolysis pathway**. Inverse cross-validated Correct Classification Rates (CCR) for all models for the Glycolysis pathway for *Arabidopsis*.

The glycolysis experiment results are displayed in Figure 5. Figure 5 presents the mean correct classification rates (CCR) for HME3M and comparison methods for each pathway dataset built from the three trailed gene activity tolerances. The number of mixture components *M *is varied from 2 to 10. It is clear from Figure 5 that for all tolerances the mean CCR for HME3M after *M *= 2 is consistently greater than all other methods and the optimal performance being observed at *M *= 4. An interesting feature of Figure 5 is that after the optimal performance has been reached, the addition of more components seems to not affect the overall classification accuracy. This shows HME3M to be resistant to overfitting and complements the results of the noise simulation experiments in Table 3.

**Figure 6:**
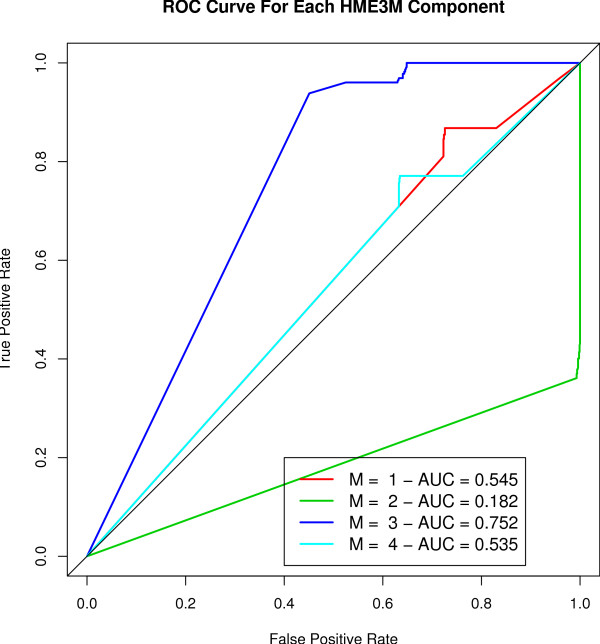
**ROC curve of all paths for the optimal model (*M *= 4) for the glycolysis pathway**.

**Figure 7:**
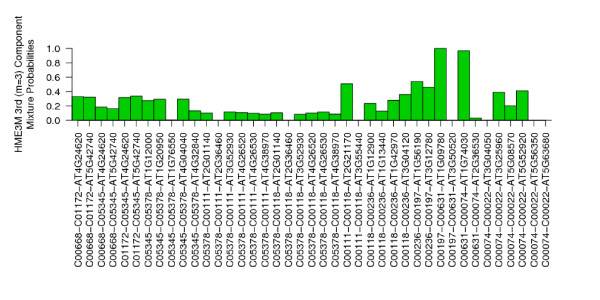
**Transition probabilities for the most expressed glycolysis path (*m *= 3) that separates flowers from rosette for Arabidopsis**. Training set CCR = 0.818, AUC = 0.752.

The ROC curves for each HME3M component are presented in Figure 6 and clearly show that the third component is the most important with an AUC of 0.752, whereas the other three components seem to hold limited or no predictive power. A bar plot of the HME3M transition probabilities (*θ*_*m*_) for the third (*m *= 3) component is presented in Figure 7. Overlaying the transition probabilities from Figure 7 onto the full network in Figure 3 it is found that for three transitions only single genes are required for the reaction to proceed:

• 

• 

A further analysis of the genes identified reveals the interaction between AT1G09780 (*θ *= 1) and AT1G74030 (*θ *= 0.969) is of particular importance in stress response of *Arabidopsis*. A literature search on these genes identified both AT1G09780 and AT1G74030 as important in the response of *Arabidopsis *to environmental stresses such as cold exposure, salt and osmotic stress [15,16]. However, AT2G21180, apart from being involved in glycolysis, has not previously been found to be strongly involved in any specific biological function. Interestingly however, a search of TAIR [17] revealed that AT2G21180 is found to be expressed in the same growth and developmental stages as well as in the same plant structure categories as both AT1G09780 and AT1G74030. These findings are indicative of a possible relationship between these three genes in particular in the response to environmental stress.

The second path connecting compounds C00197 through C00631 to C00074 is found by HME3M to have a high probability of being differently expressed when comparing glycolysis in flowers and rosette leaves. The branching of glycolysis at Glycerate-3P (*C*00197) through to Phosphoenol-Pyruvate (*C*00074) corresponds known variants of the glycolysis pathway in *Arabidopis*; the glycolysis I pathway located in the cytosol and the glycolysis II pathway located in the plastids [17]. The key precursor that leads to the branching within cytosol variant by the reactions to convert Beta-D-Fructose-6P (*C*05378) to Beta-D-Fructose-1,6P (*C*05378) using diphosphate rather than ATP [17]. Referencing the included pathway genes in Figure 7 within the reference Arabidopsis database TAIR [17] we observe that the genes specific to the percursor reactions for the cytosol variant of glycolysis are included within the pathway, i.e. the genes [*AT*1*G*12000, *AT*1*G*20950, *AT*4*G*0404] for converting beta-D-fructose-6P (*C*005345) into beta-D-fructose-1,6P2 (*C*005378) utilizing diphosphate rather than ATP. HME3M's identification of the plant cytosol variant of the glycolysis pathway confirms this pathway as a flower specific, because the plastids variant is clearly more specific to rosette leaves due to their role in photosynthesis.

### KEGG Arabidopsis Pentose Phosphate Pathway

**Figure 8:**
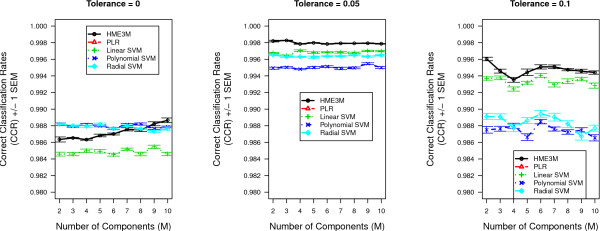
**Arabidopsis Performance results for the pentose phosphate pathway for classifying oxidative stress pathways**.

The classification performance rates for all methods to classify oxidative stress and control pathways within the pentose phosphate pathway for each tolerance level are presented in Figure 8. It is clearly observed from Figure 8 for tolerance levels 0.05 and 0.1 HME3M is outperforming all comparison models for all values of *M*. However for tolerance 0 we initially observe the polynomial and radial SVM kernels outperforming both HME3M and linear SVM. However as *M *increases we observe the performance of HME3M to steadily increase and finally after *M *= 9 HME3M is slightly outperforming both radial and polynomial SVM. This performance profile is an indication of the degree of noise within the dataset. The number of pathways identified for a tolerance of 0 is quite large, 63002 (Table 2), and decreasing slightly this tolerance level to -0.05 is seen to double the number of pathways extracted. Therefore it is reasonable to suggest that setting a tolerance of 0 is just at the edge of the pathway structure distribution below which excessive amounts of noise pathways are extracted.

In contrast increasing the tolerance level to 0.1 we observe a decrease in the performance of HME3M as *M *is increased from *M *= 2 to *M *= 4 (Figure 8). This uncharacteristic drop in performance of HME3M is the result of insufficient variation within the pathway dataset. This assertion is supported by HME3M finding the optimum model over all datasets at tolerance of 0.05. However when the gene activity tolerance is increased to 0.1 the optimal performance observed at a tolerance of 0.05 is never reached. Therefore increasing the tolerance to 0.1 is removing important pathways are required to produce the optimal model. HME3M then attempts to compensate for this lack of variation within the pathways observed at a tolerance of 0.1 by overfitting. This overfitting then leads to the decrease in performance observed as the model complexity of HME3M is increased.

**Figure 9:**
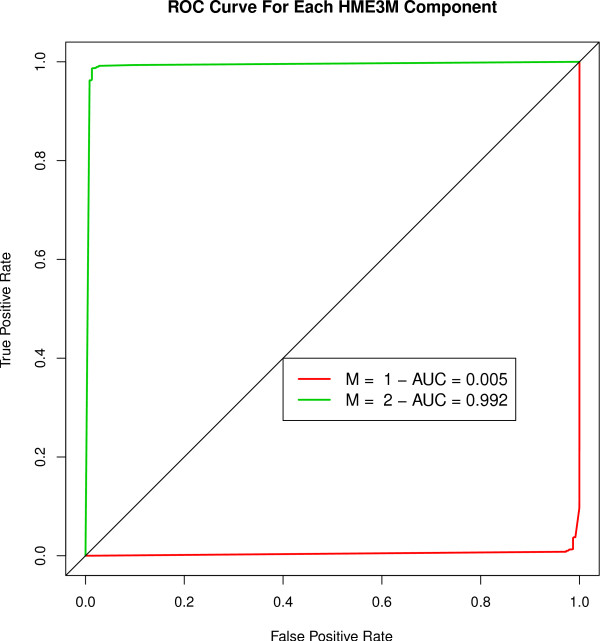
**ROC curve of all paths for the optimal model (*M *= 2) for the pentose phosphate pathway for classifying oxidative stress pathways**.

From Figure 9 we observe that the ROC curves for the optimal HME3M model (*M *= 2 tolerance = 0.05) clearly indicate one path for the oxidative label and another path for the control label. An interesting property of the ROC curves of each path is that the structure of *m *= 1 is almost exactly opposite to *m *= 2. The cause of this inverse similarity between the ROC curves is that a similar path is identified by each 3M component (*θ*_*m *= 1 _and *θ*_*m *= 2 _are correlated at *r *= 0.52) for both *m *= 1 and *m *= 2 but the signs of the PLR coefficients within each expert are flipped. In Table 4 we show the distribution of signs of the PLR coefficients for each of the two components. From Table 4 we see that for all cases when *β*_*m *= 1 _< 0 there is a 45% chance that the sign of the PLR coefficent is positive in path *m *= 2. The high correlation between the estimated pathway structure indicates that the same path is being found for both *m *= 1 and *m *= 2. However the flipping of the signs within the PLR coefficients changes the structure of *m *= 1 to predict the control label when the oxidative path in component *m *= 2 is not observed. The pathway duplication indicates that the main structure within the dataset is the activated oxidative pathway observed when *Arabidopsis *is under stress and the control group contains mainly noise pathways with little unique structure.

**Table 4:** Observed sign differences in HME3M PLR coefficients *β *for each path identified in the Pentose Phosphate pathway.

	*β*_*m *= 2 _< 0	*β*_*m *= 2 _= 0	*β*_*m *= 2 _> 0
*β*_*m *= 1 _< 0	28	2	25

*β*_*m *= 1 _= 0	1	6	0

*β*_*m *= 1 _> 0	3	0	15

**Figure 10:**
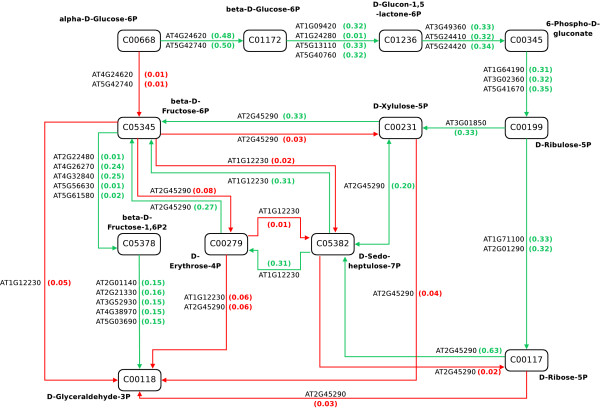
**Transition probabilities for the most expressed pentose phosphate path (*m *= 2) for Arabidopsis under oxidative stress**. The numbers in brackets represent the probability of each edge *θ*_*m *_for *m *= 2.

To visualize the oxidative class pathway we overlay the transition probabilities onto the pentose phosphate network (Figure 4) and clearly see the oxidative branch from C00668 to C00117 (D-Ribose-5P) is highlighted (Figure 10). The transition probabilities estimated by HME3M confirm the observations of [14] and show that when *Arabidopsis *is under oxidative stress the pentose phostphate pathway is clearly coordinated to produce D-Ribose-5P. However we observe that no single gene transitions can define the pathway but a coordinated set of genes that determine the path taken when the pentose phosphate cycle is subjected to oxidative stress.

## Conclusions

In this paper we have presented a novel approach for the detection of dominant pathways within a network structure for binary classification using the Markov mixture of experts model, HME3M. Simulations clearly show HME3M to outperform both PLR and SVM with linear, polynomial and radial basis kernels. When applied to actual metabolic networks with real microarray data HME3M not only maintained its superior performance but also produced biologically meaningful results.

Naturally it would be interesting to explore the performance of HME3M in other contexts where the properties of the datasets and networks are different. Future work on HME3M could be to assess the performance of different pathway activity definitions, other than simply over expressed genes. Furthermore, the 3M component of HME3M is also able to be extended to include other gene information such as protein class and function. Incorporating additional information on specific gene functions or using different pathway definitions would allow HME3M to examine metabolic pathways at several resolutions and help improve the understanding of the underlying dynamics of the metabolic network.

## Methods

### Hierarchical Mixture of Experts (HME)

A HME is an ensemble method for predicting the response where each model in the ensemble is weighted by probabilities estimated from a hierarchical framework of mixture models [18]. Our model is the simplest two level HME, where at the top is a mixture model to find clusters within the dataset, and at the bottom are the experts, weighted in the direction of each mixing component, used to classify a response. Given a response variable *y *and predictor variables *x*, a 2-layer HME has the following form,(1)

where *β*_*m *_are the parameters of each expert and *θ*_*m *_are the parameters of mixture component *m*. A HME does not restrict the source of the mixture weights *p*(*m*|*x*, *θ*_*m*_) and as such can be generated from any model that returns posterior component probabilities for the observations. Taking advantage of this flexibility we propose a HME as a method to supervise the Markov mixture model for metabolic pathways 3M [9]. Combining HME with a Markov mixture model first employs the Markov mixture to find dominant pathways. Posterior probabilities are then assigned to each sequence based on its similarity to the dominant pathway. These are then passed as input weights into the parameter estimation procedure within the supervised technique. Using the posterior probabilities of 3M to weight the parameter estimation of each supervised technique is in effect localizing each expert to summarize the predictive capability of each dominant pathway. Therefore incorporating the 3M Markov mixture model within a HME is creating a method capable of combining network structures with standard data table information. We now formally state the base 3M model and provide the detail of our proposed model, Hierarchical Mixture Experts 3M (HME3M) classifier.

### 3M Mixture of Markov Chains

The 3M Markov mixture model assumes that pathway sequences can be represented with a mixture of first order Markov chains [9]. The full model form spanning *M *components estimating the probabilities of *T *transitions is,(2)

where *π*_*m *_is the mixture model component probability, *p*(*c*_1_|*θ*_1*m*_) is the probability of the initial state *c*_1_, and *p*(*c*_*t*_, *x*_*t*_|*c*_*t*-1_, *θ*_*tm*_) is the probability of a path traversing the edge *x*_*t *_linking states *c*_*t*-1 _and *c*_*t*_. The 3M model is simply a mixture model and as such its parameters are conveniently estimated by an EM algorithm [9]. The result of 3M is *M *mixture components, where each component, *m*, corresponds to a first order Markov model defined by *θ*_*m *_= {*θ*_1*m*_, [*θ*_2*m*_, ..., *θ*_*tm*_, ..., *θ*_*Tm*_]} which are the estimated probabilities for each transition along the *m*^*th *^dominant path.

### HME3M

The HME model combining 3M and a supervised technique for predicting a response vector *y *can be achieved by using the 3M mixture probabilities *p*(*m*|*x*, *θ*_*m*_) (2), for the HME mixture component probabilities in (1). This yields the HME3M likelihood,(3)

The parameters of (3) can be estimated using the EM algorithm by defining the esponsibilities variable *h*_*im *_to be the probability that a sequence *i *belongs to component *m*, given *x*, *θ*_*m*_, *β*_*m *_and *y*. These parameters are iteratively optimized with the following E and M steps:

**E-Step: **Define the responsibilities *h*_*im*_:(4)

**M-Step: **Estimate the Markov mixture and expert model parameters:

#### (1) Estimate the mixture parameters

where *δ *(*x*_*it *_= 1) denotes whether a transition *t *is active within observation *i*, or *x*_*it *_= 1. This condition enforces the constraint that the probabilities of each set of transitions between any two states must sum to one. Additionally it can be shown that for this model all initial state probabilities *p*(*c*_1_|*θ*_1*m*_) = 1.

#### (2) Estimate the expert parameters

Using a weighted logistic regression for each expert,(6)

The original implementation of HME estimates the expert parameters, *β*_*m*_, with the Iterative Reweighted Least Squares (IRLS) algorithm, where the HME weights, *h*_*im *_are included multiplicatively by further reweighting the standard IRLS weights [10]. The IRLS iterations are Newton-Raphson steps with normal equations defined by,(7)

where  is the vector of probabilities  and *W*_*m *_is a diagonal matrix of weights such that  and *z*_*m *_is the *working *response for the IRLS algorithm . However, in this setting, *X *is a sparse matrix of binary pathways where we expect and are explicitly looking for dominant pathways. Thus, simple IRLS maximization of (6) is likely to be inaccurate. Furthermore, the severity of the sparsity within *X *is compounded by the additional weighting required by the experts' inclusion into the HME architecture. These conditions will manifest themselves in duplicate rows within *X*, causing rank deficiency and results in unstable estimates for the parameters of a logistic regression model. Therefore the simple IRLS scheme proposed by [10] is inappropriate for use in this case. To overcome the rank deficiency issue we propose using a regularized form of logistic regression [19].

### Penalized logistic regression (PLR)

Penalized Logistic Regression (PLR) uses a penalty [20] to allow for the coefficients of logistic regression to be run over a sparse or large dataset. In this paper the use of PLR is necessary to overcome the rank deficient nature of the data matrix and allow for stable estimation of the HME3M parameters. PLR maximizes *β*_*m *_subject to a ridge penalization |*β*_*m*_|^2 ^controlled by *λ *∈ [0, 2],(8)

The size of *λ *directly affects the size of the estimates for *β*_*m*_. As *λ *approaches 2 the estimates for *β*_*m *_will become more sparse, and as *λ *approaches 0 the estimates for *β*_*m *_approach the IRLS estimates. In this case we choose the ridge penalty for reasons of computational simplicity. The ridge penalty allows the regularization to be easily included within the estimation by a simple modification to the Netwon-Raphson steps (7). The Iterative Reweighted Ridge Regression (IRRR) equations are given by,(9)

where Λ is a *P *× *P *diagonal matrix with *λ *along the diagonal where *P *is the number of variables in *X *and *z*_*m *_is the working response as specified in (7).

However, another issue is that the Iterative Reweighted Least Squares algorithm (IRLS) used for estimating the parameters of a PLR is known to be unstable and not guaranteed to converge [20].

Furthermore our personal experience of IRLS in the HME context indicates the need for additional control over the rate of learning of the experts. This experience suggests that if the PLR iterations converge too quickly the estimates of *β*_*m *_reach a local optimum. A subsequent effect is the HME likelihood in the following iterations becomes erratic as the EM responsibilities (4) are dominated by the PLR probabilities *p*(*y*|*x*, *β*_*m*_) which do not necessarily reflect the structure within the 3M parameters. The different rates of convergence between the 3M and PLR parameters can cause instabilities in the HME3M likelihood. This problem has been noted by [18] and a solution is proposed by the imposition of a learning rate on the gradient descent form of the IRLS algorithm. This gradient descent method ensures that at each iteration, a step will be taken to maximize *β*_*m*_, a sufficient condition for the EM algorithm. However this method allows for control of the learning rate of the experts by the imposition of a learning penalty *α *∈ [0, 1] on the coefficient updates. The parameter update for gradient descent PLR regularization is then computed by:(10)

where Λ is a diagonal matrix with the regularization parameter *λ *along the diagonal and *W*_*m *_is a diagonal matrix of observation weights combining information from the IRLS algorithm and the HME architecture. The observation weights are defined to be , where  weights the observations to optimally predict *y *by  sourced from the IRLS algorithm, and *h*_*im *_are the EM responsibilities (4). This update for *β*_*m *_gives control over the size of the coefficients through *λ *and speed in which these parameters are learned through *α*. It is noted by [18] that this method will converge to the same solution as the IRLS method, however the effect of *α *will increase the number of iterations for convergence. In (10) the action of *λ *is to control the size of each *β*_*m *_by artificially inflating their variance.

## Competing interests

The authors declare that they have no competing interests.

## Authors' contributions

TH and HM developed the method and conceived the experimental designs. TH implemented the method and performed the experiments. All authors read and approved the final manuscript.
